# Organ donation after extracorporeal cardiopulmonary resuscitation: a nationwide retrospective cohort study

**DOI:** 10.1186/s13054-024-04949-5

**Published:** 2024-05-13

**Authors:** Tetsuya Yumoto, Kohei Tsukahara, Takafumi Obara, Takashi Hongo, Tsuyoshi Nojima, Hiromichi Naito, Atsunori Nakao

**Affiliations:** Department of Emergency, Critical Care, and Disaster Medicine, Faculty of Medicine, Dentistry, and Pharmaceutical Sciences, Okayama University, 2-5-1 Shikata-cho, Kita-ku, Okayama, 700-8558 Japan

**Keywords:** Brain death, Cardiopulmonary resuscitation, Extracorporeal membrane oxygenation, Organ transplantation, Out-of-hospital cardiac arrest, Tissue and organ procurement

## Abstract

**Background:**

Limited data are available on organ donation practices and recipient outcomes, particularly when comparing donors who experienced cardiac arrest and received extracorporeal cardiopulmonary resuscitation (ECPR) followed by veno-arterial extracorporeal membrane oxygenation (ECMO) decannulation, versus those who experienced cardiac arrest without receiving ECPR. This study aims to explore organ donation practices and outcomes post-ECPR to enhance our understanding of the donation potential after cardiac arrest.

**Methods:**

We conducted a nationwide retrospective cohort study using data from the Japan Organ Transplant Network database, covering all deceased organ donors between July 17, 2010, and August 31, 2022. We included donors who experienced at least one episode of cardiac arrest. During the study period, patients undergoing ECMO treatment were not eligible for a legal diagnosis of brain death. We compared the timeframes associated with each donor’s management and the long-term graft outcomes of recipients between ECPR and non-ECPR groups.

**Results:**

Among 370 brain death donors with an episode of cardiac arrest, 26 (7.0%) received ECPR and 344 (93.0%) did not; the majority were due to out-of-hospital cardiac arrests. The median duration of veno-arterial ECMO support after ECPR was 3 days. Patients in the ECPR group had significantly longer intervals from admission to organ procurement compared to those not receiving ECPR (13 vs. 9 days, *P* = 0.005). Lung graft survival rates were significantly lower in the ECPR group (log-rank test *P* = 0.009), with no significant differences in other organ graft survival rates. Of 160 circulatory death donors with an episode of cardiac arrest, 27 (16.9%) received ECPR and 133 (83.1%) did not. Time intervals from admission to organ procurement following circulatory death and graft survival showed no significant differences between ECPR and non-ECPR groups. The number of organs donated was similar between the ECPR and non-ECPR groups, regardless of brain or circulatory death.

**Conclusions:**

This nationwide study reveals that lung graft survival was lower in recipients from ECPR-treated donors, highlighting the need for targeted research and protocol adjustments in post-ECPR organ donation.

**Supplementary Information:**

The online version contains supplementary material available at 10.1186/s13054-024-04949-5.

## Background

A worldwide crisis in organ shortage is intensifying as the need for transplantations spikes; however, the supply of available organs falls short of meeting this escalating demand, further widening the gap between those in need and the organs available [1]. In response, the significance of comprehensive screening for brain death in the intensive care unit (ICU), particularly following cardiac arrest, to identify potential organ donors has been increasingly emphasized [2, 3].

Extracorporeal cardiopulmonary resuscitation (ECPR) for out-of-hospital cardiac arrest (OHCA) has been increasingly employed as an emerging rescue treatment strategy [4, 5]. However, the implementation of ECPR introduces complex ethical challenges, primarily because it frequently results in patients being placed on mechanical support with minimal prospects of neurological recovery [6]. Previous research has indicated that patients resuscitated with ECPR exhibit a markedly higher rate of brain death compared to those who undergo conventional CPR [2]. Indeed, a large retrospective study of ECPR in Japan, a leading country in the field of ECPR, revealed that decisions to withhold or withdraw life-sustaining therapy were most frequently made on the first day, with a median decision time of 2 days following admission to the ICU. Importantly, the perceived unfavorable neurological prognosis was the primary reason for the withhold or withdraw life-sustaining therapy decision [7]. In Japan, the legal diagnosis of brain death while on extracorporeal membrane oxygenation (ECMO) was not allowed until recent guideline amendments [8]. Further, the actual practice patterns and prevalence of organ donation following ECMO discontinuation have not been thoroughly investigated. A registry study from Europe suggests that organ donation rates are higher in patients undergoing ECPR than those receiving conventional CPR, indicating a potential for increasing organ donations through ECPR [9, 10].

This situation highlights the need for a comprehensive investigation into the practices of organ donation following ECPR, encompassing donor characteristics and the impact on recipients. To date, there has been a lack of research focused on the outcomes for recipients of organs from donors who have undergone ECPR, as well as those who have not. This study aims to fill this gap by examining the current practices and outcomes of organ donation post-ECPR, thereby enhancing our understanding of the potential for organ donation following cardiac arrest.

## Methods

### Study design and ethics

This was a nationwide retrospective cohort study in Japan using the Japan Organ Transplant Network database, covering the entire cohort of deceased organ donors of any ages, from July 17, 2010 through August 31, 2022. The Japan Organ Transplant Network prospectively collects data, including basic patient information and the clinical course details. These are recorded in a paper-based format by a transplant coordinator, based upon the patient’s medical records. This study was approved by the Ethics Committee of the Japan Organ Transplant Network (Approved Number: 15) and the Ethics Committee of Okayama University Hospital (Approved Number: K2303-030). Informed consent from the patient's family or legal representative was waived in this study.

### Organ donation policy in Japan

The history and details of organ donation policy and the surrounding system are elaborated on elsewhere [11, 12]. To summarize, Japan's organ donation policy after brain death underwent significant revision with the implementation of the revised Organ Transplant Law on July 17, 2010. This revision introduced two major changes: firstly, it established a system permitting organ donation with only the family's consent when the preferences of the deceased are unknown; and secondly, it authorized the transplantation of organs from children under 15 years of age. Prior to these changes, organ donation after brain death was permitted only if the patient had formally documented their wish to donate their organs. As a direct consequence of these policy revisions, the number of organ transplants from brain-dead donors saw a substantial increase, from 86 cases recorded between 1997 and 2010 to 413 cases between 2010 and 2017. In Japan, the donor's family has the right to choose which organs can be procured for recipients.

### Protocol for organ donation following brain death

The timing of presenting organ donation as a potential end-of-life care option is entirely at the discretion of the attending physician or according to hospital policy. Per the Japan Organ Transplant Network procedures, this option is typically presented after the clinical confirmation of brain death. However, if a patient is considered a potential organ donor due to devastating brain damage, the presentation of this option can proceed before the confirmation of brain death. Upon family consent, the process requires two distinct legal confirmations of brain death, conducted at least 6 h apart (or 24 h for children under 6 years old), through comprehensive neurological tests, an apnea test, and electroencephalography, leading to the eventual retrieval of organs. Previously, during the study period, individuals undergoing ECMO treatment were not eligible for a legal diagnosis of brain death until the guidelines were updated on January 1, 2024. Therefore, during our study, brain death could only be diagnosed post-decannulation of veno-arterial (VA) ECMO, when possible. In Japan, the transfer of potential organ donors between hospitals for the purpose of donation is prohibited.

### Protocol for organ donation following circulatory death

In Japan, controlled donation after circulatory death programs, particularly those using VA ECMO perfusion for organ preservation, have not been widely introduced [13]. Consequently, only kidneys and pancreases are typically donated after circulatory death under the scenario of unexpected circulatory demise. Accordingly, all donors after circulatory death have been categorized as IIb or VI, in accordance with the modified Maastricht classification [14]. The placement of a catheter for organ perfusion and the administration of heparin are permitted only after a diagnosis of brain death has been confirmed and the donor's family has consented to preoperative procedures. This allows for the placement of a catheter before cardiac arrest and the administration of heparin.

### Study population and data extraction

The data source for this study was the Japan Organ Transplant Network database. We included all deceased organ donors from whom at least one organ was recovered and subsequently transplanted. From this cohort, we specifically selected those individuals who had experienced at least one episode of cardiac arrest either before or after hospital arrival were selected. This selection was based on the free text comments that summarized the clinical course from admission to the legal determination of brain death. We received the anonymized data as follows: whether the donation was after brain death or circulatory death, age, sex, primary disease or injury, the modified Maastricht classification for donation after circulatory death (either IIb or VI as mentioned above), time intervals from admission to brain death confirmation, presentation of the option for organ donation, legal determination of brain death, organ procurement, the number of organs donated, and, if applicable, the duration of VA ECMO use in patients who received ECPR. The matched data from donors and recipients, provided by the Japan Organ Transplant Network using identifiable numbers, were used to observe graft survival rates over the longest follow-up period.

### Outcomes

The primary outcome was timeframe for the organ donation process, spanning from admission to organ procurement. Secondary outcomes included the number of organs donated and their graft survival rates.

### Statistical analysis

Continuous data were expressed as medians with interquartile range (IQR), and categorical data as counts and percentages. Patients were stratified based on whether they underwent ECPR and the type of donation (either after brain death or circulatory death) for comparative analyses. Comparisons between the two groups employed the Mann–Whitney U test for continuous variables and the Chi-square test for categorical variables. Graft survival curves were generated using the Kaplan–Meier method and were compared with the log-rank test. Graft survival is defined as the graft still functioning and not having been rejected by the recipient's body at a specified time post-transplantation. This excludes cases where the patient has been relisted for transplantation. Specifically for kidney transplants, graft survival also includes the period until the patient becomes dependent on dialysis again. Donor and recipient characteristics were not matched between groups. In an exploratory analysis, as ECMO technology and management have developed over last years, graft survival rates except for small intestine were compared between the periods from 2010 to 2017 and 2018 to 2022. Missing data were removed during the analysis whenever comparisons were made. All tests were two-tailed, and a *P* value of < 0.05 was considered statistically significant. Analyses were conducted using Prism 10.0.3 (GraphPad, San Diego, CA) and IBM SPSS Statistics 26 (IBM SPSS, Chicago, IL).

## Results

During the study period, there were 370 donors after brain death with an episode of cardiac arrest, of which 26 (7.0%) patients received ECPR and 344 (93.0%) did not receive ECPR. Additionally, there were 160 donors after circulatory death, among whom 27 (16.9%) patients received ECPR and 133 (83.1%) did not.

### Donation after brain death

Table 1 shows the demographic and clinical characteristics of donors after brain death with an episode of cardiac arrest, revealing similar basic demographics between groups. However, the majority of cases in the ECPR group were of cardiac origin. The median duration of VA ECMO support in the ECPR group was 3 days (IQR, 1 to 4). Compared to those not receiving ECPR, patients in the ECPR group experienced significantly longer intervals from admission to the presentation of the organ donation option to their families (5 vs. 3 days, *P* = 0.012), to the clinical confirmation of brain death (9 vs. 5 days, *P* = 0.001), and to organ procurement (13 vs. 9 days, *P* = 0.005).

**Table 1:** The demographic and clinical characteristics of the donors after brain death

	(+) ECPR n = 26	(−) ECPR n = 344	*P* value
Age, median (IQR), y	43 (33, 51)	44 (30, 55)	0.779
Under 18 years old, n (%)	2 (8)	32 (9)	0.565
Male sex, n (%)	17 (65)	211 (61)	0.835
Body mass index	21.8 (18.3, 24.0)	22.5 (19.7, 25.0)	0.294
Cardiac arrest after hospital arrival, n (%)	0 (0)	8 (2)	0.618
Maximum duration of cardiac arrest, median (IQR), min	56 (42, 78)	42 (26, 60)	0.045
Missing, n (%)	14 (54)	77 (22)	
Minimum duration of cardiac arrest, median (IQR), min	52 (42, 63)	26 (18, 36)	< 0.001
Missing, n (%)	6 (23)	43 (18)	
Primary disease or injury, n (%)			< 0.001
Hanging	0 (0)	105 (31)	
Subarachnoid hemorrhage	0 (0)	90 (26)	
Asphyxia due to foreign body	0 (0)	32 (9)	
Traumatic brain injury	0 (0)	21 (6)	
Intracerebral hemorrhage	0 (0)	16 (5)	
Drowning	1 (4)	15 (4)	
Fatal arrhythmia	10 (39)	8 (2)	
Acute myocardial infarction	6 (23)	7 (2)	
Carbon monoxide poisoning	0 (0)	6 (2)	
Asthma attack	0 (0)	5 (2)	
Drug intoxication	0 (0)	4 (1)	
Pulmonary embolism	3 (12)	3 (1)	
Traumatic asphyxia	0 (0)	3 (1)	
Hemorrhagic shock	0 (0)	2 (1)	
Accidental hypothermia	2 (8)	0 (0)	
Others	3 (12)	13 (4)	
Unknown	1 (4)	14 (4)	
Year of organ procurement, n (%)			0.869
2010-2014	4 (15)	66 (19)	
2015-2018	11 (42)	132 (38)	
2019-2022	11 (42)	146 (42)	
Duration of VA ECMO support, median (IQR), days	3 (1, 4)		
Missing, n (%)	10 (38)		
Time from admission to present the option of organ donation to the family, median (IQR), days	5 (3, 9)	3 (1, 6)	0.012
Missing, n (%)	4 (15)	95 (28)	
Time from admission to brain death confirmation, median (IQR), days	9 (5, 14)	5 (3, 8)	0.001
Missing, n (%)	0 (0)	6 (2)	
Time from admission to the first legal diagnosis of brain death, median (IQR), days	11 (7, 18)	7 (4, 11)	0.007
Time from admission to the second legal diagnosis of brain death, median (IQR), days	12 (7, 20)	8 (5, 12)	0.007
Time from admission to organ procurement, median (IQR), days	13 (9, 21)	9 (6, 14)	0.005

*ECPR* extracorporeal cardiopulmonary resuscitation, *IQR* interquartile range, *VA* veno-arterial

Table 2 presents the number and distribution of organs donated, comparing the ECPR and non-ECPR groups. The median number of organs donated was similar between the groups (5 vs. 5, *P* = 0.294). The proportion of heart donations was significantly lower in the ECPR group compared to the non-ECPR group (50% vs. 80%, *P* < 0.001). However, the donation rates for other organs were comparable between the two groups.

**Table 2:** The number and distribution of organs donated after brain death between ECPR and non-ECPR groups

	(+) ECPR n = 26	(−) ECPR n = 344	*P* value
Number of organs donated, median (IQR)	5 (4, 6)	5 (4, 6)	0.294
Heart, n (%)	13 (50)	275 (80)	< 0.001
Lung, n (%)	16 (62)	229 (67)	0.601
Right	6 (23)	78 (23)	0.962
Left	5 (19)	80 (23)	0.638
Bilateral	9 (35)	131 (38)	0.726
Liver, n (%)	24 (92)	301 (88)	0.470
Whole	23 (89)	278 (81)	0.320
Split	1 (4)	23 (7)	0.571
Split (the other portion)	1 (4)	23 (7)	0.571
Pancreas, n (%)	14 (54)	181 (53)	0.904
Kidney, n (%)	25 (96)	308 (90)	0.279
Right	24 (92)	301 (88)	0.470
Left	24 (92)	301 (88)	0.470
Bilateral	1 (4)	4 (1)	0.254
Small intestine, n (%)	1 (4)	6 (2)	0.449

*ECPR* extracorporeal cardiopulmonary resuscitation, *IQR* interquartile range

**Fig. 1:**
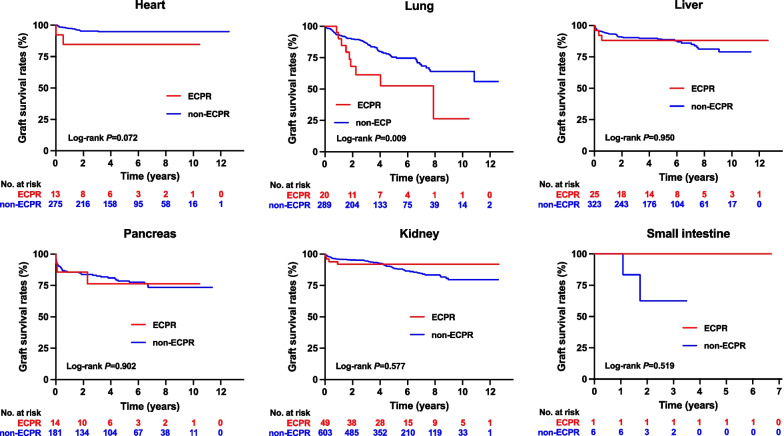
The Kaplan–Meier curve of graft survival for each organ among recipients from brain-dead organ donors, comparing those who had received ECPR with those who had not. The *P* values obtained from the log-rank test for heart, lung, liver, pancreas, kidney, and small intestine were 0.072, 0.009, 0.950, 0.902, 0.577, and 0.519, respectively. The median observation periods for grafts from donors who experienced cardiac arrest and received ECPR versus those from non-ECPR donors, respectively, were as follows: for heart grafts, 1203 days (IQR: 542 to 2278) and 1690 days (IQR: 908 to 2610); for lung grafts, 777 days (IQR: 573 to 1816) and 1323 days (IQR: 596 to 2211); for liver grafts, 1816 days (IQR: 671 to 2438) and 1551 days (IQR: 738 to 2466); for pancreas grafts, 1083 days (IQR: 442 to 2118) and 1708 days (IQR: 677 to 2673); for kidney grafts from brain-dead donors, 1787 days (IQR: 736 to 2429) and 1690 days (IQR: 987 to 2576); and for small intestine grafts, 2446 days (IQR: 2446 to 2446) and 703 days (IQR: 404 to 1217); ECPR: extracorporeal cardiopulmonary resuscitation

Figure 1 illustrates the graft survival curves for each organ. The lung graft survival rates were significantly lower in the ECPR group compared to the non-ECPR group (log-rank test *P* = 0.009). Graft survival rates for both unilateral (single) and bilateral (double) lung grafts among recipients from brain-dead organ donors were generally lower in the ECPR group. This reduction was statistically significant for unilateral lung grafts, as detailed in Additional file 1. No significant differences were observed in the graft survival rates of other organs.

### Donation after circulatory death

Table 3 outlines the demographic and clinical characteristics of donors post-circulatory death, highlighting a higher prevalence of male donors in the ECPR group compared to the non-ECPR group. Regarding the primary disease or injury, cardiac diseases were notably more common among ECPR patients, mirroring the trend observed in brain-dead organ donors. The reporting of the duration of VA ECMO support was limited by extensive missing data. Additionally, the intervals from admission to offering the option of organ donation to the family and proceeding to organ procurement showed no significant differences between the two groups.

**Table 3:** The demographic and clinical characteristics of the donors after circulatory death

	(+) ECPR n = 27	(−) ECPR n = 133	*P* value
Age, median (IQR), y	46 (40, 61)	48 (38, 59)	0.920
Under 18 years old, n (%)	1 (4)	1 (1)	0.208
Male sex, n (%)	21 (78)	72 (54)	0.023
Body mass index	22.3 (20.1, 24.3)	22.0 (19.3, 24.8)	0.939
Missing, n (%)	4 (15)	4 (3)	
Cardiac arrest after hospital arrival, n (%)	3 (11)	8 (6)	0.340
Maximum duration of cardiac arrest, median (IQR), min	60 (35, 74)	40 (20, 62)	0.045
Missing, n (%)	11 (41)	51 (38)	
Minimum duration of cardiac arrest, median (IQR), min	57 (41, 71)	26 (11, 36)	< 0.001
Missing, n (%)	10 (37)	43 (29)	
Primary disease or injury, n (%)			< 0.001
Subarachnoid hemorrhage	3 (11)	38 (29)	
Hanging	0 (0)	37 (28)	
Asphyxia due to foreign body	0 (0)	7 (5)	
Cervical spinal code injury	0 (0)	7 (5)	
Traumatic brain injury	0 (0)	6 (5)	
Intracerebral hemorrhage	0 (0)	5 (4)	
Drowning	0 (0)	3 (2)	
Fatal arrhythmia	6 (22)	3 (2)	
Acute myocardial infarction	8 (30)	3 (2)	
Traumatic asphyxia	0 (0)	3 (2)	
Carbon monoxide poisoning	0 (0)	2 (2)	
Asthma attack	2 (7)	1 (1)	
Drug intoxication	0 (0)	1 (1)	
Pulmonary embolism	4 (15)	1 (1)	
Hemorrhagic shock	0 (0)	1 (1)	
Others	3 (11)	6 (5)	
Unknown	1 (4)	9 (7)	
Year of organ procurement, n (%)			0.331
2010-2014	8 (30)	60 (45)	
2015-2018	12 (44)	47 (35)	
2019-2022	7 (26)	26 (20)	
Duration of VA ECMO support, median (IQR), days	3 (2, 6)		
Missing, n (%)	23 (85)		
Modified Maastricht classification, n (%)			0.001
II	23 (85)	73 (54.9)	
VI	4 (15)	60 (45.1)	
Time from admission to present the option of organ donation to the family, median (IQR), days	2 (0, 3)	3 (1, 5)	0.167
Missing, n (%)	12 (44)	79 (59)	
Time from admission to brain death confirmation, median (IQR), days	7 (2, 77)	4 (2, 6)	0.402
Missing, n (%)	23 (85)	72 (54)	
Time from admission to organ procurement, median (IQR), days	5 (2, 9)	7 (2, 12)	0.230

*ECPR* extracorporeal cardiopulmonary resuscitation, *IQR* interquartile range, *VA* veno-arterial, *ECMO* extracorporeal membrane oxygenation

^a^This number includes patients who were not diagnosed with brain death

Table 4 reports the number and distribution of organs donated, comparing the ECPR and non-ECPR groups. The pancreas was not donated in either group. High kidney donation rates were noted in both groups. Left kidney donation was lower in the ECPR group compared to non-ECPR group (85 vs. 96%, *P* = 0.023).

**Table 4:** The number and distribution of organs donated after circulatory death between ECPR and non-ECPR groups

	(+) ECPR n = 27	(−) ECPR n = 133	*P* value
Number of organs donated, median (IQR)	2 (2, 2)	2 (2, 2)	0.227
Pancreas, n (%)	0 (0)	0 (0)	N/A
Kidney, n (%)	26 (96)	130 (98)	0.661
Right	25 (93)	125 (94)	0.786
Left	23 (85)	128 (96)	0.023
Bilateral	1 (4)	0 (0)	0.026

*ECPR* extracorporeal cardiopulmonary resuscitation, *IQR* interquartile range

**Fig. 2:**
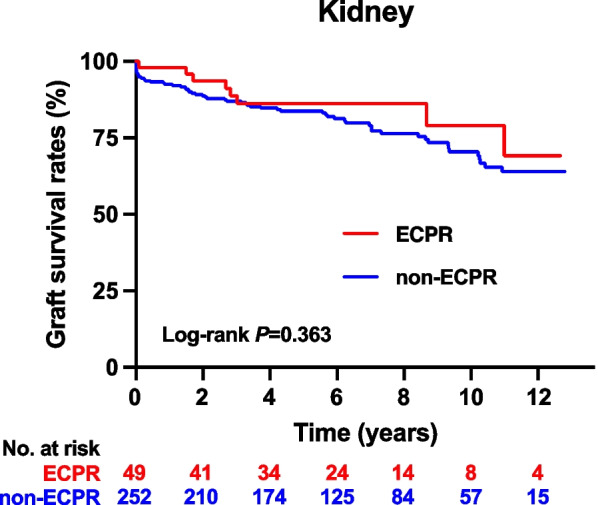
The Kaplan–Meier curve of graft survival for kidney among recipients from circulatory-dead organ donors, comparing those who had received ECPR with those who had not. The *P* values obtained from the log-rank test were 0.363. The median observation periods for grafts from donors who experienced cardiac arrest and received ECPR versus those from non-ECPR donors were 2071 days (IQR: 1004 to 3110) and 2160 days (IQR: 1175 to 3535), respectively. ECPR: extracorporeal cardiopulmonary resuscitation

Figure 2 shows the graft survival curve for kidneys, indicating no significant differences between the ECPR and non-ECPR groups.

### Exploratory analysis

Lung graft survival rates for ECPR patients from the periods 2010–2017 and 2018–2022 showed no significant difference, as indicated in Additional file 2 (log-rank test *P* = 0.827). Similarly, liver graft survival rates for the same periods did not differ significantly within the ECPR group; however, liver graft survival rates from ECPR patients were significantly lower compared to those from non-ECPR patients in 2018–2022 (log-rank test *P* = 0.023). Comparable patterns were observed in graft survival for other organs.

## Discussion

In this nationwide cohort study conducted in Japan, we found that time intervals from admission to organ procurement after brain death were significantly longer for ECPR patients compared to non-ECPR patients. However, these intervals were similar following circulatory death. The number of organs donated after either brain death or circulatory death was comparable between the ECPR and non-ECPR groups. Despite similar proportions of lung donations between groups, lung graft survival was significantly lower in recipients from brain-dead organ donors who received ECPR compared to those without ECPR.

Our findings indicate that the time from admission to organ procurement in Japan is longer than that reported in other countries [15, 16]. This variation may be attributed to the extensive discussions required around end-of-life care options, including organ donation, which are further complicated by the cultural emphasis on family involvement in medical decision-making processes [17]. Moreover, we noted that donors who underwent ECPR experienced longer delays to organ procurement compared to those who did not receive ECPR. This delay is likely due to legal constraints preventing the determination of brain death until after the decannulation of VA ECMO, highlighting a unique challenge in the organ donation process in Japan. Notably, guidelines were updated on January 1, 2024, allowing the diagnosis of brain death while on ECMO.

The influence of a donor’s ICU stay duration on recipient outcomes remains underexplored. A study from Germany indicated that the ICU stay duration of donors did not significantly impact the survival rates or outcomes following heart transplantation [18]. Similarly, another study concluded that the duration of a donor's ICU stay had no significant effect on patient and graft survival rates after pediatric liver transplantation [19]. These insights suggest that the ICU stay duration may not critically affect transplantation outcomes, consistent with our observations, with the possible exception of lung transplants. Meanwhile, there is limited data on donors who are brain dead with ongoing ECMO support. Among the available studies, the largest, conducted in France, focused predominantly on donors who received VA ECMO. It revealed that kidneys procured and transplanted from these donors did not exhibit differences in survival and functional outcomes compared to those from donors who were brain dead without ECMO support [20].

ECPR is typically administered to patients with a potential or presumed cardiac origin [4, 5]. Consequently, even after the successful decannulation of VA ECMO, we observed a significantly lower rate of heart donations in the ECPR group compared to the non-ECPR group. Meanwhile, despite similar lung donation rates between groups, lung graft survival was significantly lower in ECPR recipients from brain-dead donors than in those without ECPR. This trend was consistent across both time periods, from 2010 to 2017 and from 2018 to 2022. This phenomenon may be attributable to “ECMO lung”, a condition characterized by lung injury induced by VA ECMO, resulting from inflammatory injury or pulmonary congestion [21]. Meanwhile, we observed that liver graft survival rates from ECPR patients were significantly lower compared to those from non-ECPR patients in 2018–2022. Although we could not fully explain the reasons, this might be partly due to severe cardiovascular condition affecting liver function through mechanisms such as cardiac hepatopathy, which includes impaired arterial perfusion and passive congestion from elevated venous pressure, often exacerbated by the hemodynamic instability and changes in liver perfusion associated with ECMO support [22].

According to the study using Japanese Diagnosis Procedure Combination Database, the prevalence of ECPR for OHCA from July 2010 to March 2017 was 2.6% (5612/212,295) [23]. Over the past 12 years, despite the lack of legal permission of brain death diagnosis during ECMO support, our study identified 53 deceased organ donors who had undergone VA ECMO due to at least one episode of cardiac arrest, with the vast majority experiencing OHCA. The exact number of brain death cases among patients who received ECPR for OHCA in the prior study is unknown; however, considering a meta-analysis indicating a 27.9% prevalence of brain death following ECPR [6], it can be speculated that the majority may have died without the opportunity for organ donation.

This study has several limitations. First, regarding donor characteristics, the study did not capture donors' comorbidities, the detailed processes involved in organ donation, or outcomes focused on the donors' families. Second, the analysis was limited by the absence of specific data, particularly the duration of VA ECMO support. These missing data restricted our ability to analyze and adjust graft survival outcomes in relation to the duration of ECMO support. Third, from the perspective of recipients, essential characteristics, including factors known to influence graft survival such as human leukocyte antigen mismatches and primary or underlying diseases, were unavailable. As a result, these variables were not adjusted for in our analysis [24–26]. Lastly, detailed recipient data was not available, and the small sample size precluded matching between groups, further constraining our analysis.

Despite these limitations, our research provides crucial insights into the patterns of organ donation and long-term graft survival after ECPR, based on extensive nationwide data. Although diagnosing brain death while on ECMO is now permitted in Japan, scenarios in which brain death is diagnosed after successful decannulation of ECMO are expected to increase as the use of ECPR as a strategy for OHCA expands worldwide. While the primary goal of ECPR should not be organ donation, our findings underline the necessity for additional research to develop thorough guidelines for end-of-life care and the organ donation process in such scenarios. Additionally, our study suggests that lung transplantation from donors who underwent ECPR may result in worse graft survival compared to those who did not receive ECPR. This aspect, as well as the impact on other organs, warrants further investigation in future research.

## Conclusions

In this nationwide study from Japan, we discovered that lung graft survival was lower in recipients from ECPR-treated donors. These results emphasize the influence of ECPR on organ donation and underscore the need for further research to refine end-of-life care and organ donation protocols, particularly concerning lung graft survival and its effects on other organs following ECPR.

### Supplementary Information

**Additional file 1**. The Kaplan–Meier curves comparing survival of unilateral (single) and bilateral (double) lung grafts among recipients from brain-dead organ donors, categorized by whether they received ECPR or not. The *P* values from the log-rank test for unilateral and bilateral lung graft survival were 0.049 and 0.104, respectively. The median observation periods for grafts from donors who experienced cardiac arrest and received ECPR versus those from non-ECPR donors, respectively, were as follows: for unilateral lung, 817 days (IQR: 553 to 1816) and 1311 days (IQR: 582 to 2196); and for bilateral lung, 707 days (IQR: 535 to 2174) and 1374 days (IQR: 695 to 2261). ECPR: extracorporeal cardiopulmonary resuscitation.

**Additional file 2**. The Kaplan–Meier curves comparing survival of heart, lung, liver, pancreas, and kidney (both from donation after brain death and donation after circulatory death) grafts, according to two periods: 2010–2017 and 2018–2022. The left set of curves represents comparisons between ECPR and non-ECPR groups from 2010 to 2017. The middle set of curves shows comparisons between ECPR and non-ECPR groups from 2018 to 2022. The right set of curves compares the two time periods among patients who received ECPR. ECPR: extracorporeal cardiopulmonary resuscitation, DBD: donation after brain death, DCD: donation after circulatory death.

## Data Availability

Data not available due to ethical restrictions.

## Abbreviations

DBD: Donation after brain death
DCD: Donation after circulatory death
ECMO: Extracorporeal membrane oxygenation
ECPR: Extracorporeal cardiopulmonary resuscitation
IQR: Interquartile range
OHCA: Out-of-hospital cardiac arrest
VA: Veno-arterial
