# Supplementary figures and images for: Organ donation after extracorporeal cardiopulmonary resuscitation: a nationwide retrospective cohort study
Source: Crit Care. 2024 May 13;28:160. doi: 10.1186/s13054-024-04949-5 (PMC11092201; doi:10.1186/s13054-024-04949-5)

## Slide 1
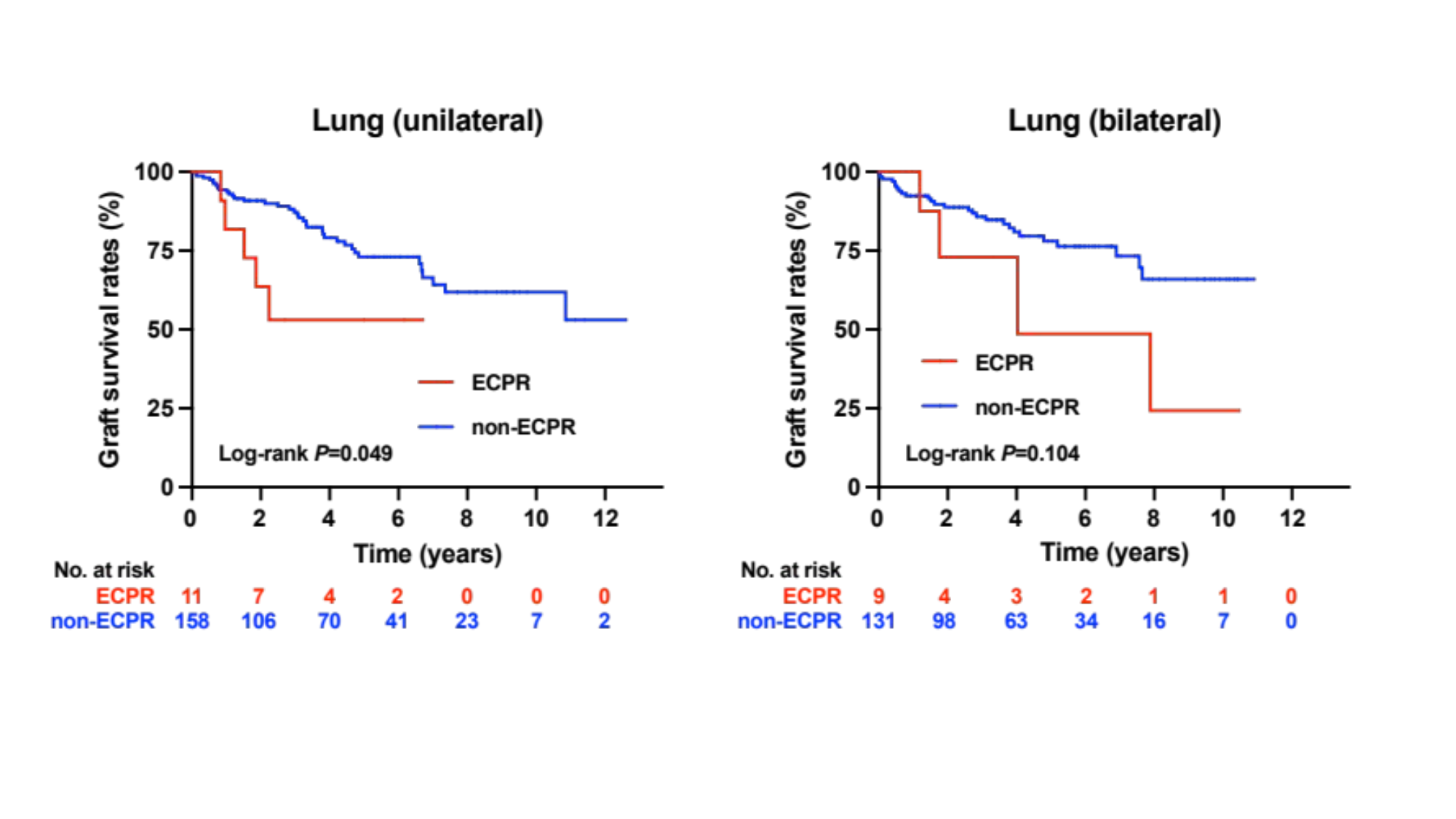

Supplement: Supplementary file 1 — Additional file 1. The Kaplan–Meier curves comparing survival of unilateral (single) and bilateral (double) lung grafts among recipients from brain-dead organ donors, categorized by whether they received ECPR or not. The P values from the log-rank test for unilateral and bilateral lung graft survival were 0.049 and 0.104, respectively. The median observation periods for grafts from donors who experienced cardiac arrest and received ECPR versus those from non-ECPR donors, respectively, were as follows: for unilateral lung, 817 days (IQR: 553 to 1816) and 1311 days (IQR: 582 to 2196); and for bilateral lung, 707 days (IQR: 535 to 2174) and 1374 days (IQR: 695 to 2261). ECPR: extracorporeal cardiopulmonary resuscitation. [file 13054_2024_4949_MOESM1_ESM.pptx]

## Slide 1
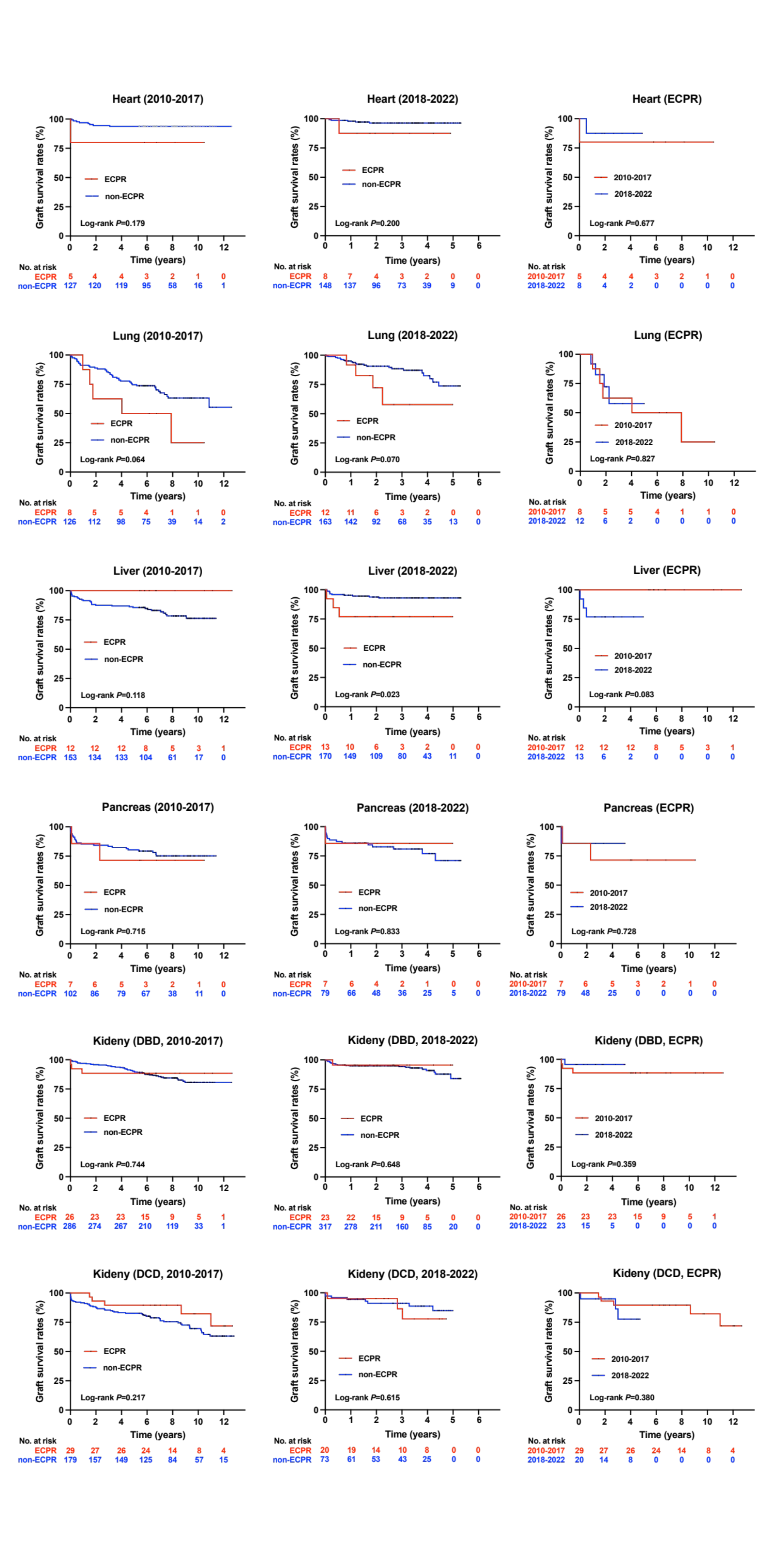

Supplement: Supplementary file 2 — Additional file 2. The Kaplan–Meier curves comparing survival of heart, lung, liver, pancreas, and kidney (both from donation after brain death and donation after circulatory death) grafts, according to two periods: 2010–2017 and 2018–2022. The left set of curves represents comparisons between ECPR and non-ECPR groups from 2010 to 2017. The middle set of curves shows comparisons between ECPR and non-ECPR groups from 2018 to 2022. The right set of curves compares the two time periods among patients who received ECPR. ECPR: extracorporeal cardiopulmonary resuscitation, DBD: donation after brain death, DCD: donation after circulatory death. [file 13054_2024_4949_MOESM2_ESM.pptx]
